# Supplementary figures and images for: Divergent Metabolomic Signatures of TGFβ2 and TNFα in the Induction of Retinal Epithelial-Mesenchymal Transition
Source: Metabolites. 2023 Jan 31;13(2):213. doi: 10.3390/metabo13020213 (PMC9966219; doi:10.3390/metabo13020213)

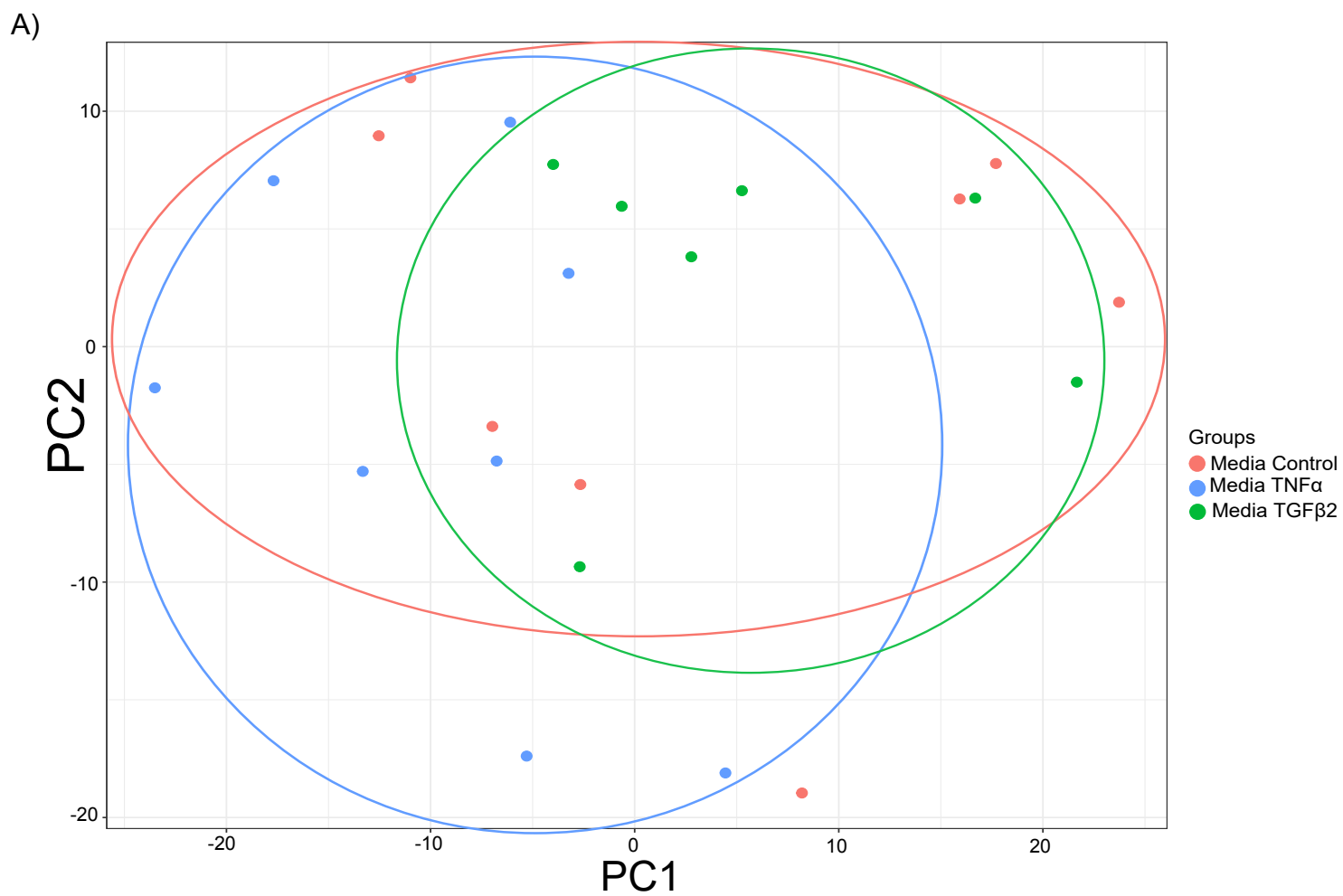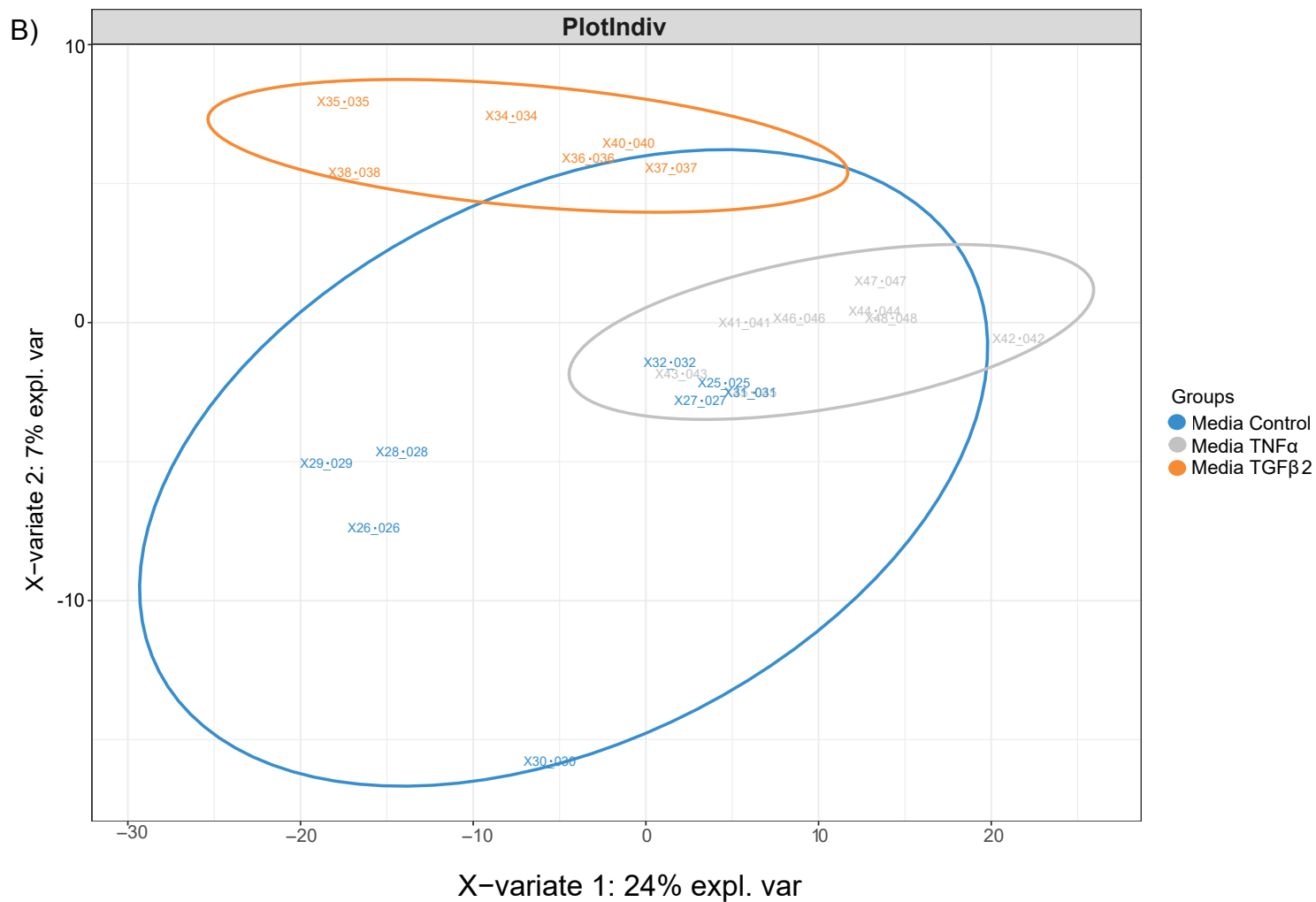

Supplement: Supplementary file 1 [file metabolites-13-00213-s001.zip › Figure S1.pdf]

A)

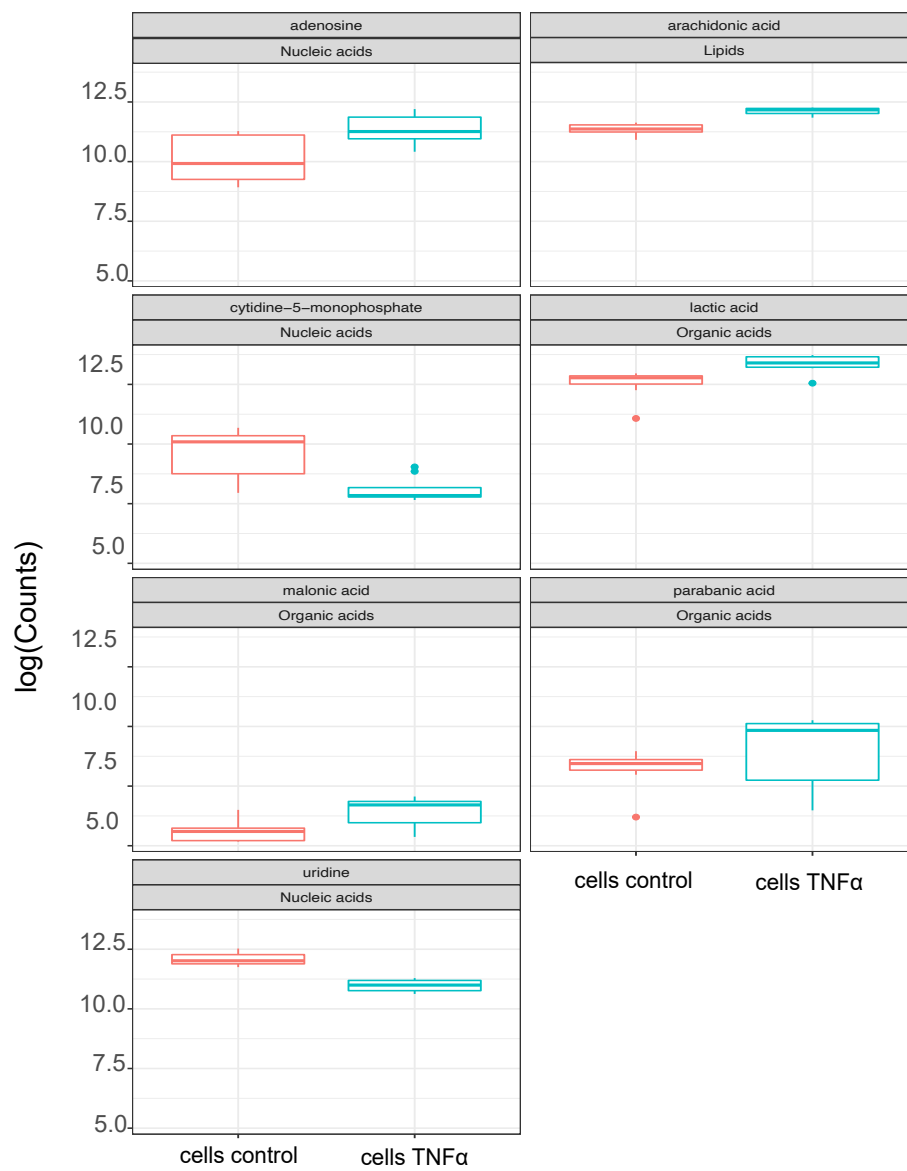

Treatment

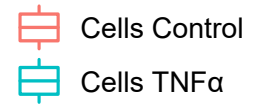

B)

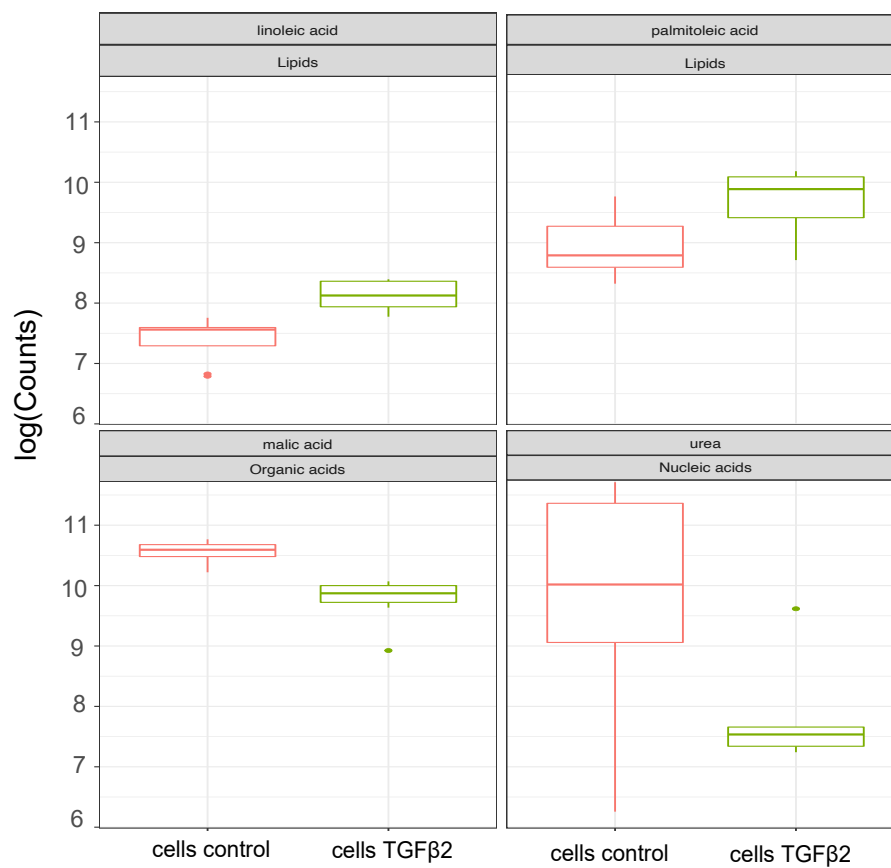

Treatment

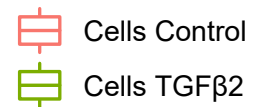

Supplement: Supplementary file 1 [file metabolites-13-00213-s001.zip › Figure S2.pdf]

### TNF $\alpha$ -treated H-RPE cells vs control

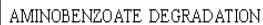

Supplement: Supplementary file 1 [file metabolites-13-00213-s001.zip › Figure S3.pdf]

## TGFβ2-treated H-RPE cells vs control

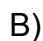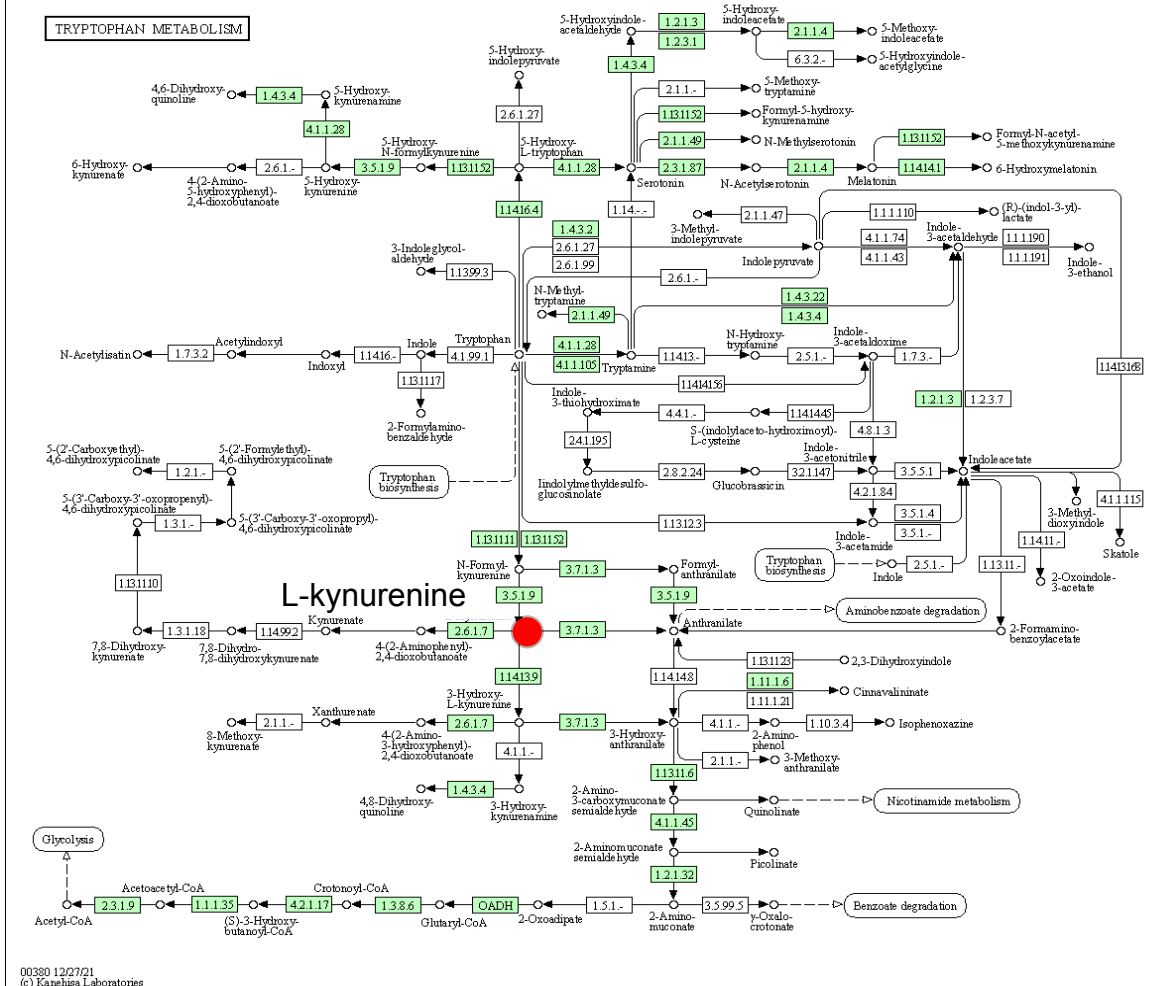

Supplement: Supplementary file 1 [file metabolites-13-00213-s001.zip › Figure S4.pdf]
